# An Unusual Occurrence of Tamoxifen-Induced Maculopathy in a Young Woman With Hormone Receptor-Positive Post-mastectomy Breast Carcinoma

**DOI:** 10.7759/cureus.64545

**Published:** 2024-07-14

**Authors:** Deeksha Jawale, Priyanka Bhoj, Amitabh Pandagle, Ankit Sharma, Asmita Kulshrestha

**Affiliations:** 1 Department of Radiation Oncology, Cama and Albless Hospital, Mumbai, IND; 2 Department of Surgical Oncology, Tata Memorial Hospital, Mumbai, IND

**Keywords:** chemotherapy, modified radical mastectomy (mrm), maculopathy, tamoxifen, breast cancer

## Abstract

Tamoxifen, a selective estrogen receptor modulator (SERM), is a hormone therapy used for the treatment of estrogen receptor (ER)-positive breast cancer. We report the case of a 29-year-old premenopausal lady with a history of infertility treatments who was diagnosed with ER-positive infiltrating ductal carcinoma (IDC) of the breast. Following a modified radical mastectomy (MRM) and adjuvant systemic chemotherapy, tamoxifen was recommended as part of her adjuvant hormonal therapy.

After over three years of tamoxifen use, the patient complained of gradual blurring of vision in both eyes. Ophthalmological examinations indicated bilateral maculopathy, a rare but alarming ocular side effect attributed to tamoxifen use.

This case report emphasizes the significance of ophthalmic tests in patients on tamoxifen therapy to monitor any potential ocular side effects. While tamoxifen has shown remarkable benefits in the adjuvant treatment of ER-positive breast cancer, including lowering the chance of recurrence and increasing survival rates, clinicians must be acquainted with rare but potential vision-threatening consequences such as tamoxifen-induced maculopathy. Early detection and timely management are critical in reducing the risk of vision loss in patients receiving tamoxifen for breast cancer.

## Introduction

Tamoxifen is a selective estrogen receptor modulator (SERM) used for the treatment of breast cancer. In 1962, it was initially developed to be used as a contraceptive. However, in the 1980s, it underwent a transformative journey when clinical trials revealed its efficacy in treating estrogen receptor (ER)-positive breast cancer [1]. Tamoxifen is now a cornerstone of adjuvant hormonal therapy for women with breast cancer. It is often prescribed for a period of five to 10 years.

Tamoxifen has demonstrated proven benefit in prolonging disease-free survival among breast cancer patients. It also reduces recurrence in ER-positive and node-negative (stages I and II) breast cancers across all age groups [2]. Additionally, tamoxifen use has been approved as a chemo-preventive agent in women who have a higher risk of developing invasive and non-invasive breast cancer (factors increasing risk are strong family history, breast cancer gene (BRCA) mutation, atypical hyperplasia in breast tissue and high-density mammogram) [3].

We present a case of a young woman on tamoxifen who developed visual side effects to show the importance of conducting an ophthalmic evaluation before starting tamoxifen therapy and regular monitoring for any possible ocular effects.

## Case presentation

A 29-year-old premenopausal patient presented with complaints of a painless lump in the right breast. She was nulliparous and had taken infertility treatments with clomiphene citrate three years back. A tru-cut biopsy of the lump was suggestive of high-grade intraductal carcinoma, following which the patient underwent a right modified radical mastectomy (MRM) along with right axillary lymph node dissection.

Post-operative histopathological examination revealed a 2x1.8x1.3 cubic cm tumor located in the upper outer quadrant. All 13 dissected axillary lymph nodes were negative for malignancy. Margins remained free, and no lymphovascular and perineural invasion were identified, with the skin and nipple-areolar complex also being free of the tumor. Her diagnosis was then confirmed as right infiltrating ductal carcinoma, with Nottingham's histological score being 7, suggesting moderate differentiation. The tumor was grade three on the modified Bloom-Richardson scale and staged as IIA (pT2N0Mx). The immunohistochemistry molecular profile was ER-positive (Allred score 3/8), progesterone receptor, and Her2/neu negative.

The general examination was unremarkable. On local examination, the right chest wall and axilla post-operative scar sites were healthy. No abnormality was detected in the left breast and axilla. All routine blood investigations were found to be within normal limits. Chest X-ray and ultrasonography of the abdomen and pelvis were unremarkable for any distant metastasis.

Following surgery, she received six cycles of adjuvant systemic chemotherapy with cyclophosphamide (600 mg/m^2^), Adriamycin (60 mg/m^2^), and 5-fluorouracil (600 mg/m^2^) every three weeks. She then received external beam radiotherapy (EBRT) using a breast board for immobilization with arms above the head and six megavoltage photon beam energy on a linear accelerator (LINAC) with three-dimensional conformal radiation therapy (3D-CRT) technique. The total dose given was 40 Gray in 15 fractions to the right chest wall and supraclavicular fossa over 23 days in accordance with the hypofractionation dosage protocol. The course of therapy was uneventful.

**Figure 1:**
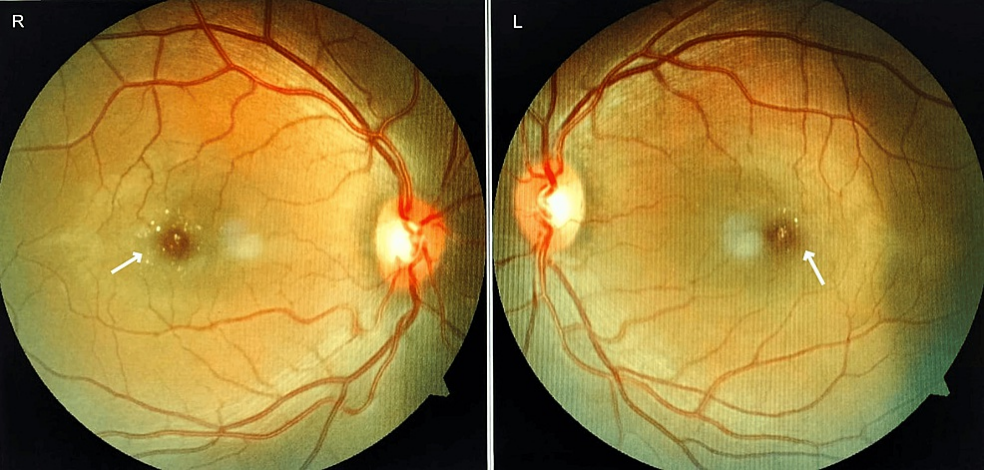
Fundoscopy image Fundoscopy of the right (R) and left (L) eyes shows refractile crystalline deposits with pigment changes in the macula. The right eye has more deposits than the left eye. Turning off the temporal vessels nasally is seen.

**Figure 2:**
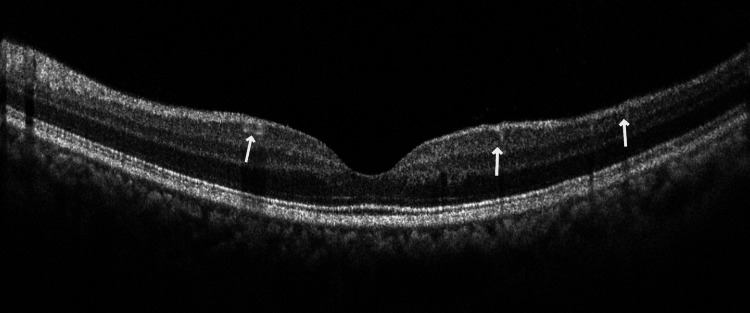
Optical coherence tomography - left eye The left eye optical coherence tomography image shows hyper-reflectivity in the inner layer of the retina. They are likely crystalline deposits and have caused backshadowing. Macular edema is absent.

**Figure 3:**
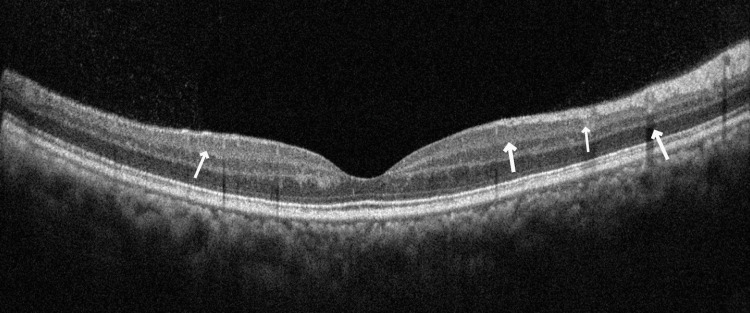
Optical coherence tomography - right eye The right eye optical coherence tomography image shows more hyper-reflective areas in the retina than the left eye. Backshadowing due to crystalline retinal deposits is present, with no cystoid macular edema.

Given her ER-positive status, she was started on adjuvant hormonal therapy with tamoxifen 20 mg to be taken daily for five years. The patient adhered to scheduled follow-ups. About three years and four months into tamoxifen therapy, she reported blurred vision in both eyes for two months. She had previously worn prescription glasses with power +7 in her left eye and +6 in her right eye. An eye examination with fundoscopy and optical coherence tomography (OCT) identified bilateral ocular maculopathy (Figures 1-3). The findings have been summarized in Table 1.

**Table 1:** Ophthalmic examination findings

	Right Eye	Left Eye
Vision with glasses	6/18	6/18
Color vision	Normal	Normal
Conjunctiva	Normal	Normal
Anterior chamber	Normal depth	Normal depth
Cornea	Bright	Bright
Pupil	Normal size, reacting to light	Normal size, reacting to light
Lens	Clear	Clear
Fundus	Disc normal	Disc normal
Macula	Crystalline maculopathy	Crystalline maculopathy

Tamoxifen-induced maculopathy was suspected, with the patient having received a total dose of 24.4 grams. It was immediately discontinued, and she was prescribed oral letrozole 2.5 mg daily for the remaining duration of hormonal therapy. A positron emission tomography (PET) scan showed no evidence of recurrence or metastasis. A year later, follow-up revealed no progression in visual complaints but no significant improvement in blurred vision either.

## Discussion

The Food and Drug Administration has approved tamoxifen for treating hormone receptor-positive, invasive, and non-invasive breast cancer post-completion of primary therapy with surgery and/or radiation to reduce the risk of recurrence and improve survival rates [4].

Tamoxifen is cardioprotective and lowers the risks of coronary artery disease, myocardial infarction, stroke, arrhythmia, pericarditis, and valvular heart disease due to its cholesterol-lowering effect [5]. A raised risk of venous thromboembolism has been reported, although the absolute risk remains low [6]. Taking tamoxifen reduces the risk of breast cancer mortality by one-third in the first 10 years following diagnosis [7]. For most, this benefit outweighs the increased risk of developing endometrial cancer [8].

A higher occurrence of cataracts has been noted in women with five or more years of tamoxifen use [9]. Incidences of ocular toxicity, even on low doses of tamoxifen, have been documented, including crystalline retinal deposits, cystoid macular edema, foveal cavitations, decreased visual acuity, and keratopathy [10,11]. Patients using high doses have experienced extensive retinal lesions, macular edema, and significant visual impairment [12]. The use of high doses of tamoxifen for breast cancer has since been discontinued.

Our patient had taken infertility treatment. While in-vitro fertilization was linked to retinal detachment, ovulation induction treatments by themselves have not been found to be significantly associated with long-term ocular adverse effects, according to a large study [13]. With an increasing incidence of breast cancer worldwide, including in women under 50 years of age, the use of tamoxifen is bound to rise [14]. OCT screenings have been recommended every six months for patients who have been on tamoxifen therapy for two years or more [15].

The most common causes of damage to the macula include age-related macular degeneration and diabetic retinopathy [16]. Symptoms of maculopathy include edema, decreased visual acuity, distorted vision, poor color vision, hemorrhages, light sensitivity, and difficulty seeing in low light levels [17,18].

## Conclusions

Although the risk of tamoxifen-induced eye toxicity is low, and maculopathy is even rarer, early detection and treatment can help prevent serious vision problems. Given the lack of research on tamoxifen-induced maculopathy, especially in younger patients, this case report highlights this relatively rare complication. It emphasizes the need for patient education and regular eye check-ups.

It is important to inform patients about the possible ocular side effects of tamoxifen and encourage them to report any vision changes as soon as they notice them. By working together, patients and healthcare providers can make sure that the benefits of tamoxifen outweigh the risks, leading to the best outcomes for women taking this medication.
